# Supplementary material for: 3D Melanoma Cocultures as Improved Models for Nanoparticle-Mediated Delivery of RNA to Tumors
Source: Cells. 2022 Mar 17;11(6):1026. doi: 10.3390/cells11061026 (PMC8946997; doi:10.3390/cells11061026)
Supplement: Supplementary file 1 [file cells-11-01026-s001.zip › cells-1542833-supplementary.pdf]

Supplementary Materials

to manuscript cells-1542833

3D melanoma co-cultures as improved models for nanoparticle-mediated delivery of RNA to tumors

by Maximilian E. A. Schäfer, Florian Keller, Jens Schumacher, Heinrich Haas, Fulvia Vascotto, Ugur Sahin, Mathias Hafner, Rüdiger Rudolf

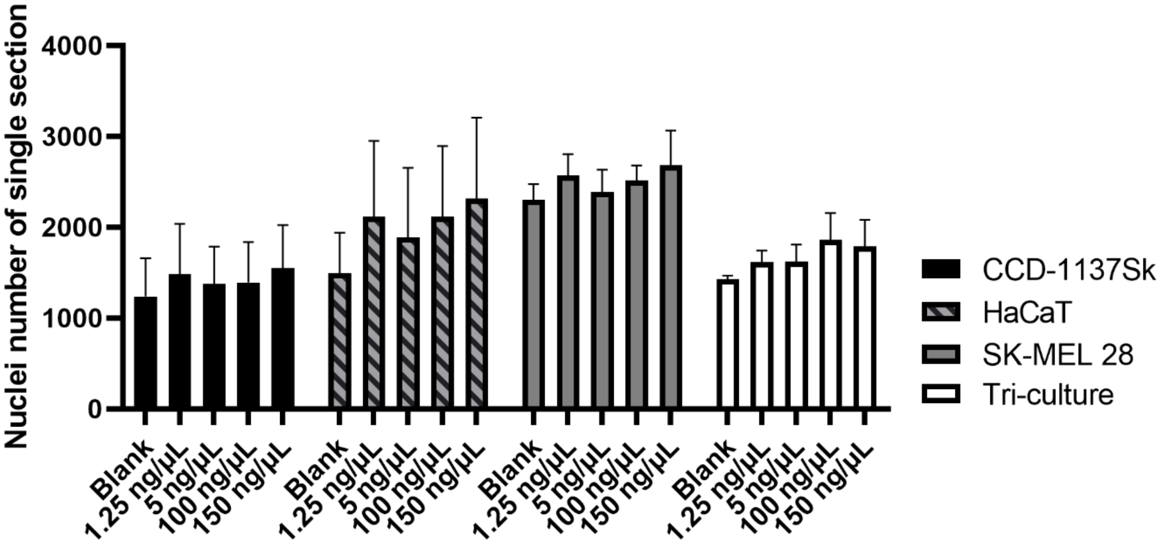

Figure S1. Number of nuclei does not vary in the absence or presence of LPX. For each culture condition, a total of 3 x 10^4 cells was seeded. Cell number and viability were determined by Vi-CELL XR. After 24 h, treatment with varying amounts of LPX loaded with eGFP coding mRNA for one day. PBS only, blank. Number of nuclei was determined by confocal microscopy of DRAQ5 signals at the end of the treatment period. Mean + SD (n=3 independent experiments). Two-way ANOVA with Tukey's multiple comparison post-hoc test shows no significant differences.

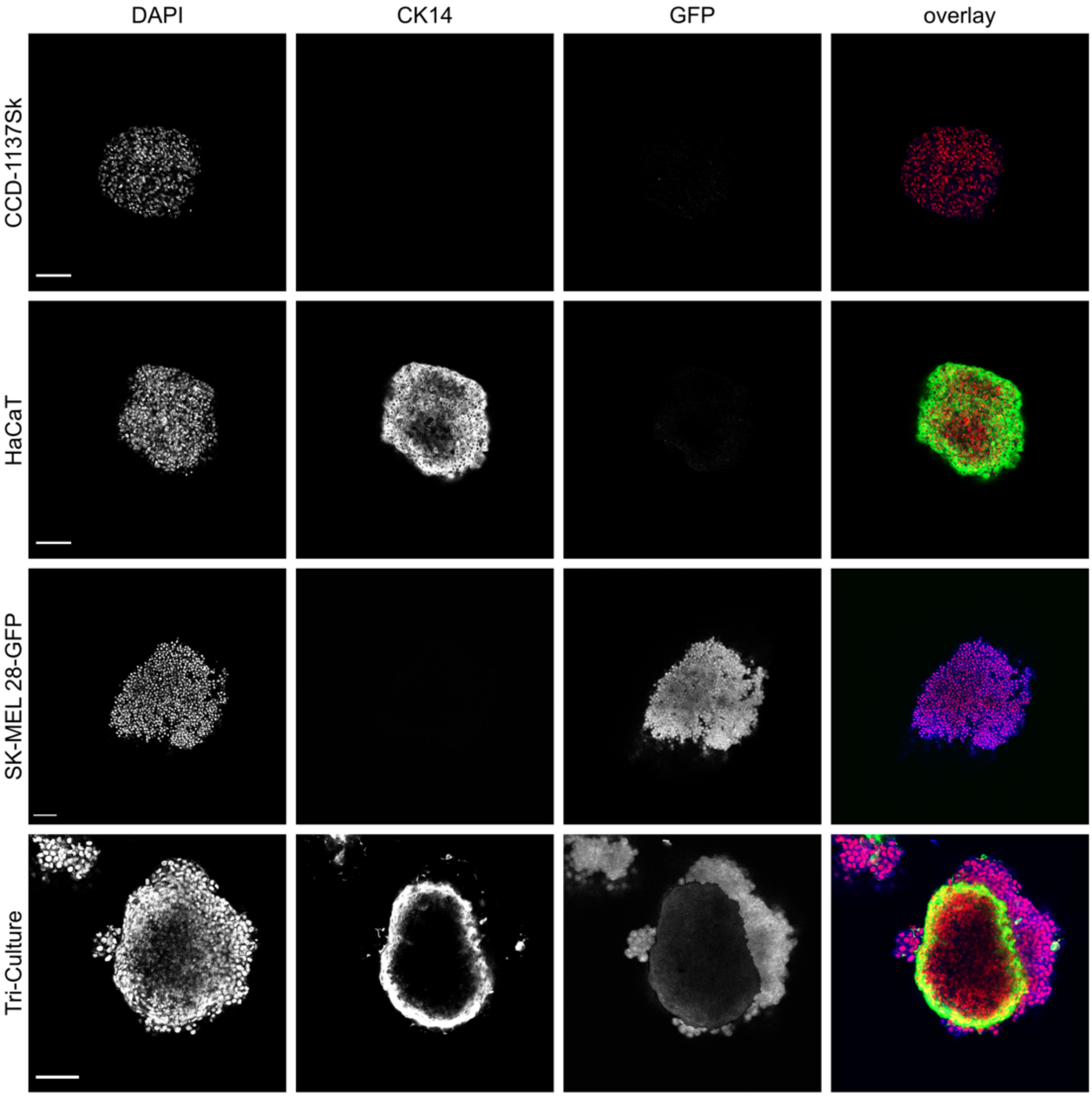

Figure S2. The use of SK-MEL 28-GFP cells and the keratinocyte-specific antibody CK14 show a defined cellular arrangement in tri-culture spheroids. Sample generation and treatment were performed as described in sections 2.1, 2.2 and 2.3. However, SK-MEL 28 spheroids and tri-culture spheroids used SK-MEL 28 cells constitutively expressing EGFP (SK-MEL 28-GFP, Angio-Proteomie, cAP-0084GFP). Spheroids were grown, fixed, labeled for nuclei with DAPI (Sigma-Aldrich, 10236276001, 1:500, red), and immunostained with anti-CK14 (Merck, MAB3232; secondary antibody Thermo Fisher Scientific, PA5-16722; both diluted 1:1,000). Panels depict single optical planes of whole mounts. Scale bars, 100 µm. According to these data and in agreement with previous findings [14,15], tri-cultures showed a core of CCD-1137Sk fibroblasts, surrounded by a HaCaT keratinocyte ring (green), and capped by SK-MEL 28-GFP melanoma cells (magenta).

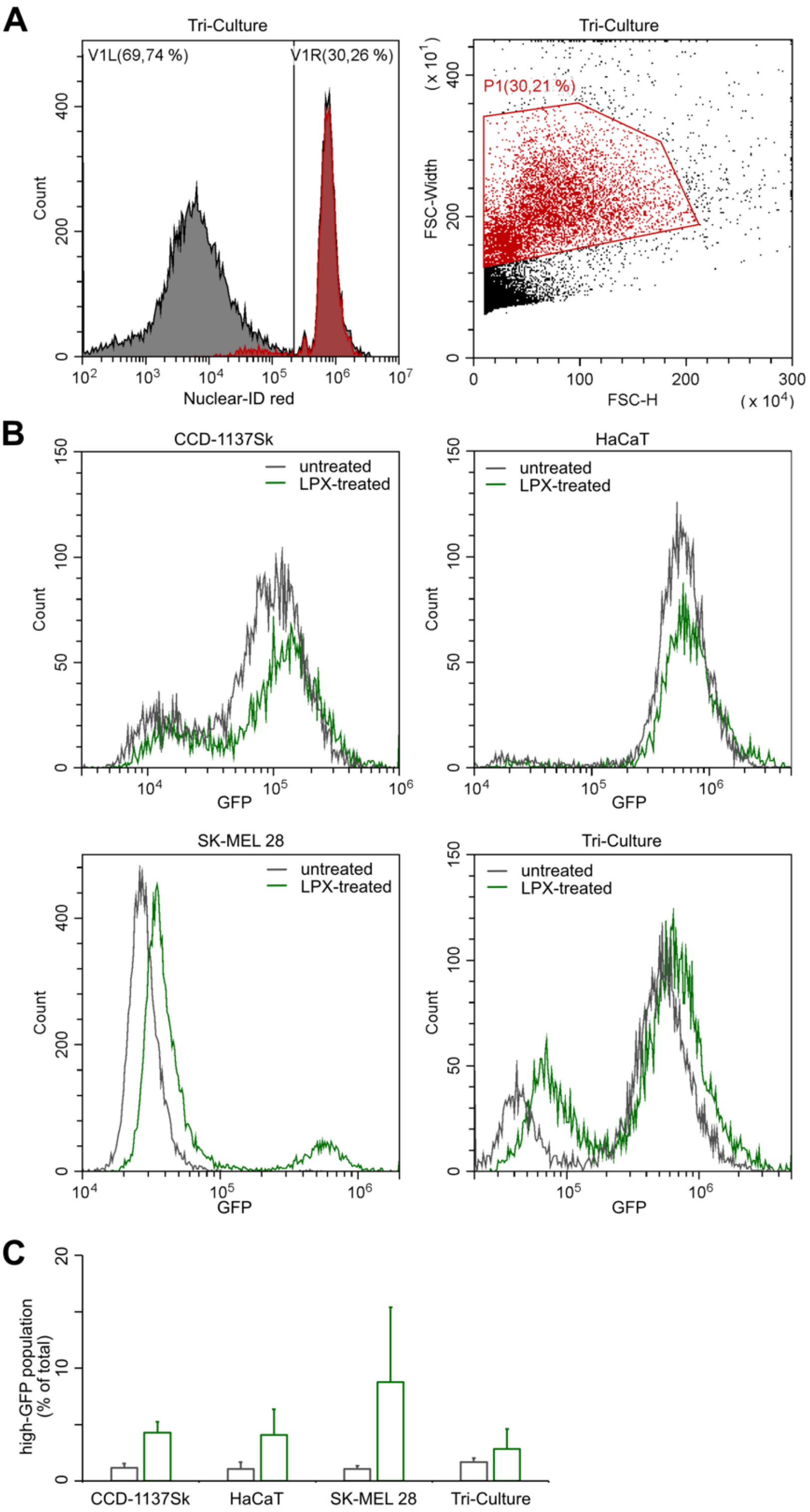

Figure S3. Cytometric analysis of LPX treated 3D mono- and co-cultures. Sample generation and treatment performed as described in sections 2.1, 2.2 and 2.3. Dissociation of the spheroids by Accumax (Invitrogen, 00-4666-56) for 30 min at RT. Stained with Nuclear-ID Red (ENZO - ENZ-52406) for 30 min, 1:250 diluted with FACS buffer (10mM HEPES, 0.5mM EDTA, 2%FCS / pH 7.2 at RT). **(A)** Exemplary blots for cytometry-settings: 20.000 ± 500 events were measured for each sample and based on a Nuclear-ID threshold only cells were included into the analysis. **(B)** Representative graphs of untreated (grey lines) and LPX-treated cultures (green lines). **(C)** Quantitative analysis of cytometry data. Graphs show the high-GFP population based on cytometry thresholds. Untreated (grey columns) and LPX-treated samples (green columns). Mean + SD. At least 96 spheroids were pooled per sample and treatment condition (n=3).

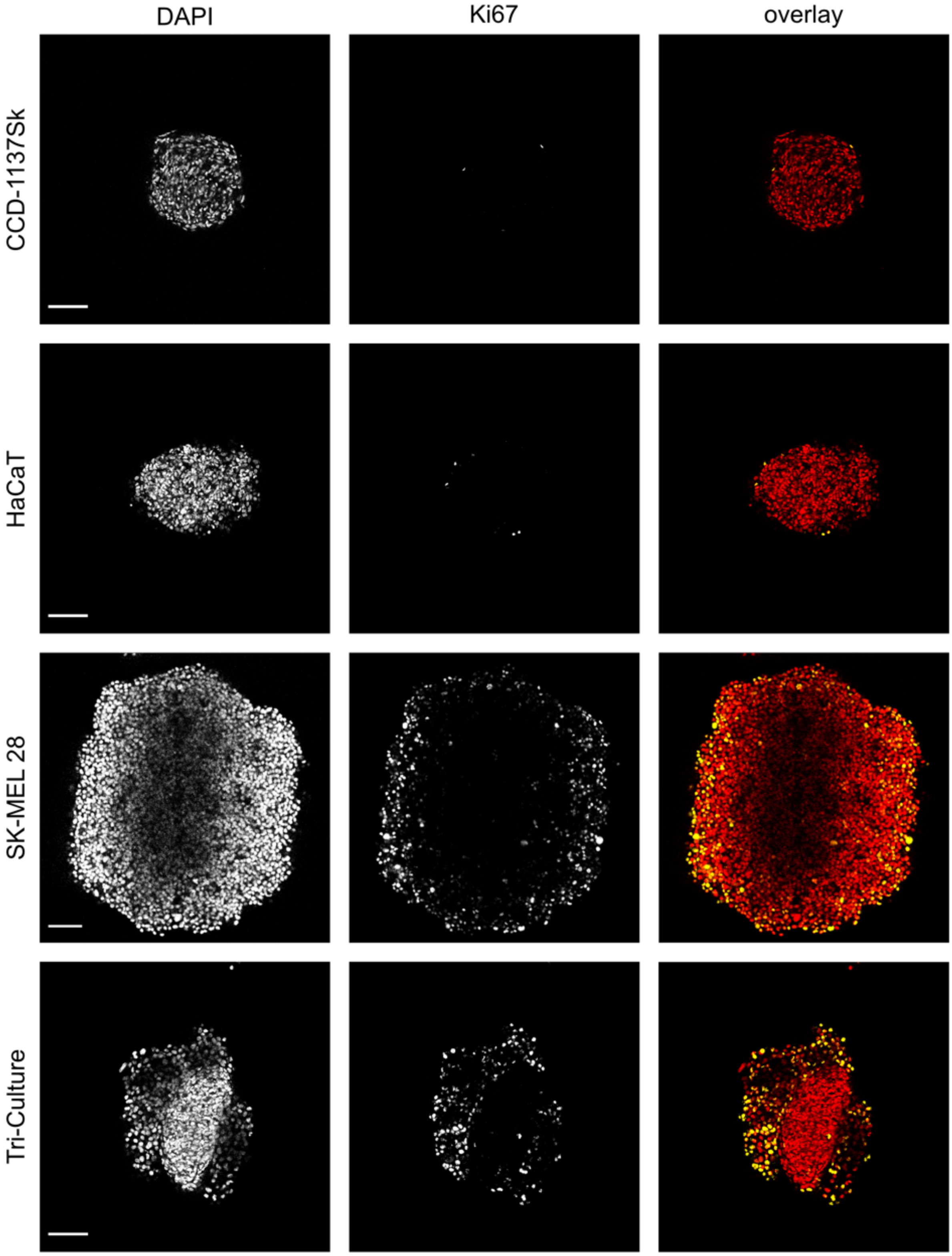

Figure S4. SK-MEL 28 cells show a higher proliferation rate in both mono- and tri-culture spheroids compared to HaCaT or CCD-1137Sk cells. Sample generation and treatment were performed as described in sections 2.1, 2.2 and 2.3. Nuclei were labeled with DAPI (Sigma-Aldrich, 10236276001, 1:500, red); the proliferation marker, Ki67, was immunostained with an anti-Ki67 antibody (Sigma-Aldrich, AB9260, 1:400, yellow) and the corresponding secondary antibody (Invitrogen, A21246, 1:1,000). All images depict single optical planes of whole mount staining. Scale bar, 100 µm.
